# Comparison between the effects of exergame intervention and traditional physical training on improving balance and fall prevention in healthy older adults: a systematic review and meta-analysis

**DOI:** 10.1186/s12984-021-00917-0

**Published:** 2021-11-24

**Authors:** Yan Chen, Yuan Zhang, Zhenxiang Guo, Dapeng Bao, Junhong Zhou

**Affiliations:** 1 Sports Coaching College, Beijing Sport University, Beijing, China; 2 Human Development and Family Sciences, University of Connecticut, Storrs, CT USA; 3 China Institute of Sport and Health Science, Beijing Sport University, Beijing, China; 4 Hebrew SeniorLife Hinda and Arthur Marcus Institute for Aging Research, Harvard Medical School, Boston, MA USA

**Keywords:** Exergame, Balance, Fall prevention, Older adults, Systematic review, Meta-analysis

## Abstract

**Objective:**

Physical training (PT, e.g., Tai Chi and strength training) has been demonstrated to improve balance control and prevent falls. Recently, exergame intervention (EI) has emerged to prevent falls by enhancing both physical and cognitive functions in older adults. Therefore, we aim to quantitatively assess and compare the effects of PT and EI on the performance of balance control and fall prevention in healthy older adults via meta-analysis.

**Methods:**

A search strategy based on the PICOS principle was used to find the publication in the databases of PubMed, EMBASE, Web of Science, Cochrane Library, and MEDLINE. The quality and risk of bias in the studies were independently assessed by two researchers.

**Results:**

Twenty studies consisting of 845 participants were included. Results suggested that as compared to PT, EI induced greater improvement in postural control (sway path length, SMD = − 0.66, 95% CI − 0.91 to − 0.41, *P* < 0.001, *I*^2^ = 0%; sway speed, SMD = − 0.49, 95% CI − 0.71 to − 0.27, *P* < 0.001, *I*^2^ = 42%) and dynamic balance (SMD = − 0.19, 95% CI − 0.35 to − 0.03, *P* = 0.02, *I*^2^ = 0%) in healthy older adults. The EI with 90–119 min/week for more than 8-week significantly reduced falls. Subgroup analyses revealed that exergames, which were designed by the two principles of repeatedly performing diversified tasks and gradually increase the difficulty of the task, induced significant effects in improving balance control and falls prevention respectively (*P* = 0.03, *P* = 0.009). In addition, intervention that combines EI and PT induced significant improvement in postural control (*P* = 0.003).

**Conclusion:**

The exergame intervention, especially the combination of EI and PT, is a promising strategy to improve balance control and reduce falls in healthy older adults. Future studies with rigorous design, larger sample size, and follow-up assessments are needed to further assess the effectiveness of diverse exergame interventions in fall prevention and to quantify the “dose-effect” relationship, as well as the carry-over effect of such intervention, which will ultimately help optimize the rehabilitative strategies to improve balance control and prevent falls.

**Supplementary Information:**

The online version contains supplementary material available at 10.1186/s12984-021-00917-0.

## Introduction

Falls are a global public health problem in the older adult population, oftentimes leading to mobility limitation, diminished quality of life, as well as increased mortality and morbidity [1–4]. One of the main factors leading to falls is the loss of balance when standing and walking [5–7]. Therefore, strategies designed to improve standing and walking performance will ultimately help reduce fall risk in older adults.

Numerous efforts have been made to improve the capacity of maintaining balance when standing and walking. Studies have shown that traditional physical training (PT), such as balance and strength training, as well as Tai Chi, can help reduce fall risks by improving the standing and walking performance [8–10]. For example, El-Khoury et al. [8] found that a 2-year balance intervention could reduce the incidence of injurious falls in older women compared to the control, and the participants showed improved balance as measured by faster time to complete the timed-up-and-go test and walk 6-m test, and longer time in single-leg stance test. However, studies also suggested that the traditional PT is time-consuming, and the training procedure is not always enjoyable for older adults, oftentimes resulting in low compliance and high drop-out rate of participants [11, 12].

Recently, exergame intervention (EI) has been implemented as a novel rehabilitative strategy for those who have cognitive-motor impairments (e.g., Parkinson and stroke) and demonstrated the great potential of enhancing balance control [13–15]. Interactive exergaming consists of a series of cognitive and motor tasks with biofeedback technology (e.g., virtual reality, step-mat, sensor) interacting with users in real-time fashion [16]. As compared to traditional PT, the biofeedback technology (e.g., virtual reality) in EI enables creating different types of the training environment and task protocol as needed, achieving a more convenient completion of intervention; and such technology provides real-time biofeedback, allowing users to adjust their motion or body movements during the training [17, 18]. More recently, several studies have shown that using EI only or EI in combination with other types of exercises could help improve balance by augmenting musculoskeletal strength, executive cognitive function, and motor control, thus helping reduce fall risk [19–21]. Stanmore et al. [21], for example, reported that as compared to using the physical exercise intervention targeting strength and balance only, a tailored 12-week of EI in combination with this type of PT (i.e., combined intervention) induced greater improvement in the performance of balance control and significantly reduced falls in people aged 55 years and older.

However, a large variance has been observed across these studies in the design of EI (e.g., EI only or EI in combination with other types of intervention) and study protocol [e.g., intervention duration, the type of control group (i.e., blank or active control)]. Such variance consequently results in inconsistent findings. For example, Bateni [22] showed that physical therapy training induced greater improvement in balance control as measured by Berg Balance Scale (BBS), when in comparison with Wii Fit training; while Chen et al. [23] showed that compared to traditional Tai-Chi exercise, reality-assisted training with selected Tai-Chi movements induced greater improvement in balance control measured by BBS, timed-up-and-go test, and functional reach test. The efficacy of EI thus remains unclear, and the underlying mechanisms through which EI influences functional performance are not fully understood. Therefore, this study aims to quantitatively analyze the effects of EI on the performance of balance control and fall prevention in older adults by completing a systematic review and meta-analysis based on the available peer-reviewed publications, with the intent to highlight recent efforts, advances, and possible avenues for future research in this important area.

## Materials and methods

### Design

This meta-analysis and systematic review was conducted following the Preferred Reporting Items for Systematic Reviews and Meta-Analyses (PRISMA), and the checklist was completed [24].

### Literature search strategy

Five electronic databases (PubMed, EMBASE, Web of Science, Cochrane Library, and MEDLINE) were used to search articles from the inception until November 13th, 2020. Only articles written in English were included. The search strategy followed the PICOS principle (Population, Intervention, Comparison, Outcome, and Study design). The following Medical Subject Headings (MeSH) terms and keywords were used for search strategy: [‘elderly’ or ‘aged’ or ‘older adults’ or ‘senior’] and [‘falls’ or ‘balance’ or ‘postural control’ or ‘motor control’] and [‘exergame’ or ‘exergaming’ or ‘fitness game’ or ‘active gaming’ or ‘Video Games’ or ‘Virtual Reality’ or ‘computer-based individual training’ or ‘interactive dynamic balance activities’ or ‘interactive dynamic balance exercises’ or ‘Simulation Training’] and (‘randomized controlled trial’ or ‘randomized’ or ‘RCT’). A manual search of the bibliographic references for extracted articles and existing reviews was conducted to identify studies not captured in the electronic searches.

### Eligibility and exclusion criteria

The inclusion criteria were: (1) the age of participants was 60 years and older; (2) the exergame intervention was used as intervention; (3) the physical training (e.g., resistance or strength training, balance training, physical therapy exercises, aerobic training, and Tai Chi) was used as control; (4) the outcomes were related to falls (i.e., number of falls, number of fallers, and fall efficacy), or balance performance (i.e., static balance, dynamic balance, and postural control); (5) the randomized controlled trial (RCT) design was applied.

The exclusion conditions included: (1) the participants were hospitalized, physically frail, or have any overt conditions that were related to diminished balance, mobility, and motor functions, including neurological disorders (e.g., stroke, Parkinson), cognitive impairments (e.g., mild cognitive impairment, dementia, Alzheimer’s disease), cardiovascular conditions (e.g., uncontrolled hypertension, heart failure, etc.), visual, vestibular, and auditory impairments, active arthritis, joint arthroplasty or fusion, any limb amputation or surgery, and psychological problem; (2) repetitive publication; (3) abstracts, system review, case report, and register trials report; (4) no comparison group; (5) non-RCT design.

### Screening process and data extraction

Titles and abstracts of studies were independently screened by two reviewers (YC and YZ) to identify studies that potentially met the inclusion criteria. The full texts of these studies were retrieved and independently assessed for eligibility by the two reviewers.

Study data were also extracted by the same researchers. The extracted data included: (1) number of falls and/or fallers during the follow-up period; (2) fall efficacy; (3) metrics to assess balance performance. We also extracted the relevant study information, including the first author, country/location, age, sample size (%female), interventions, the dosage of intervention, the comparison group, assessments, and outcome measures. Any ambiguity (e.g., the different selections or judgment of outcome measures) met by these two researchers was discussed with the help of the third reviewer (ZG). We contacted three authors for additional information, and two responded and provided numerical data that was not presented in the publication. The third study did not respond and provide the missing information.

### Quality evaluation

Two reviewers independently evaluated the included articles using the Cochrane risk-of-bias tool described in the Cochrane Handbook [25]. The contents of the article evaluation included: Random sequence generation (selection bias), Allocation concealment (selection bias), Blinding of participants and personnel (performance bias), Blinding of outcome assessment (detection bias), Incomplete outcome data (attrition bias), Selective reporting (reporting bias) and other bias. Disagreements, such as those in the criteria of risk-of-bias judgment and whether the study exists reporting bias, were solved by consensus or by consulting a third reviewer (ZG).

### Statistical analysis

RevMan 5.3 software was used to synthesize data as recommended by the Cochrane Handbook of Systematic Reviews of Interventions [25]. Continuous data were analyzed using the inverse variance approach by combining the mean difference (MD) of individual studies when the outcome was reported using the same measurement units or the standardized mean difference (SMD) of individual studies when the outcome was reported using different measurement units. Specifically, the MD was calculated as the mean difference of the outcomes in the intervention group before and after the intervention *minus* the mean difference of the outcomes in the control group before and after the intervention. The SMD was then calculated as the MD divided by the pooled intervention-specific standard deviation. For studies reporting the standard error (SE), median, maximum/minimum values, the outcomes were converted accordingly. For studies reporting effective size by subgroups, we combined the subgroup and calculated the effect size for the whole sample [25]. The magnitude of MD and SMD was classified according to the following scale: 0–0.19 represents negligible effect, 0.2–0.49 represents a small effect, 0.5–0.79 represents moderate effect, and 0.8 represents large effect [26]. *P*-value < 0.05 was considered statistically significant. The *I*^2^ statistic was used to assess the extent of heterogeneity (*I*^2^ = 0–40%, low; *I*^2^ = 30–60%, moderate; *I*^2^ = 50–90%, substantial, *I*^2^ = 75–100%, considerable) [25]. If heterogeneity was not significant (*I*^2^ < 50%), the fixed effect model was adopted. If heterogeneity was significant (*I*^2^ ≥ 50%), a random-effects model was used.

Subgroup analysis was conducted to examine the effects of different exergame types categorized following the motor learning principles of the EI design [27], different intervention types (the EI-only vs. the combined intervention), different lengths of program period (1 to 8-week vs. more than 8-week), and different weekly intervention duration (1 to 89 min, 90 to 119 min, and more than 119 min) on balance control and falls. The EI-only intervention was defined as the intervention that only included exergame, and the combined intervention was defined as the intervention that included both EI and PT. Sensitivity analysis based on effective size was also conducted, and publication bias was assessed by examining funnel plots and excluding asymmetric studies.

## Results

### Study selection

**Fig. 1:**
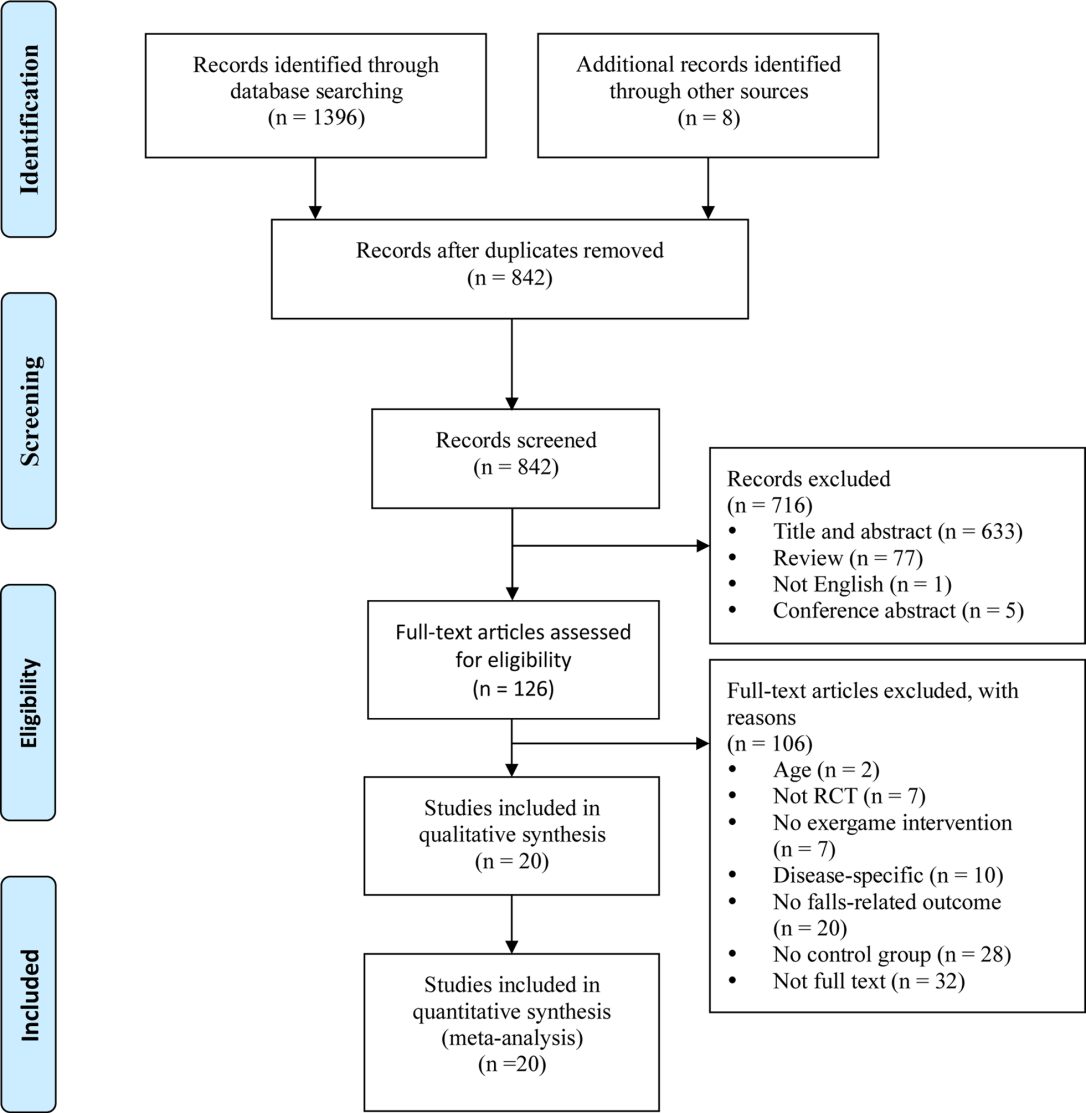
Flow chart of the study selection process

The data assessment and analysis were completed on November 13th, 2020. The systematic search yielded 1404 records: PubMed (n = 80), EMBASE (n = 53), Web of Science (n = 501), Cochrane Library (n = 407), MEDLINE (n = 355), and manual search (n = 8). 562 repetitive publications were excluded, leaving 842 articles; 716 irrelevant articles were excluded after checking inclusion and exclusion criteria; 126 articles were further excluded by reviewing the whole paper; 20 articles were then identified for inclusion in the systematic review and meta-analysis (Fig. 1).

### Characteristics of the study protocol

#### Participants

The age of all the participants was 73.39 ± 5.76 (mean ± SD) years (intervention group: 72.63 ± 5.66, control group: 73.43 ± 5.88). One study only recruited female participants [28]; one study recruited an equal number of males and females [29]; four studies recruited more males (ranging from 55 to 79.17%), while the other studies included more females (ranging from 55.56 to 100%) (Table 1). Though the participants in these studies were old adults without any major neurological diseases, they had different characteristics related to fall history. Among them, two studies recruited participants with at least one fall during the past 12-month [4, 28]; one study recruited participants without falls in the past 12-month [30]; another study recruited participants both with and without a history of falls [31], without further categorizing participants into faller group and non-faller group; the other 16 studies did not provide information about the history of falls. In addition, one study divided the exergame group into high-adherers (at least twice per week) and low-adherers (once per week) based on the number of interventions participants completed per week [32].

**Table 1:** Characteristics of participants in each study

Study	Country/location	Sample size (%female)	Age (years) Mean ± SD	Population type
Bacha [33]	Brazil	I 23 (65.2) C 23 (82.6)	I 71.0 C 66.5	Community-dwelling
Bateni [22]	USA	I 6 (50) C 6 (50)	I 68 ± 14 C 72 ± 12	Physical therapy department Community-dwelling
Bateni [22]	USA	I 6 (66.7) C 6 (50)	I 79 ± 16 C 72 ± 12	Physical therapy department Community-dwelling
Chen [23]	Taiwan	I 14 (85.7) C 14 (92.9)	I 72.2 ± 2.8 C 75.1 ± 5.5	Residents of District
Eggenberger [39]	Switzerland	I 24 (58.3) C 25 (64)	I 77.3 ± 6.3 C 80.8 ± 4.7	Local organization Residence facilities Another organization
Franco [40]	USA	I 11 (81.8) C 11 (72.7)	I 79.8 ± 4.7 C 77.9 ± 6.9	Community-dwelling
Htut [42]	Thailand	I 21 (52) C 21 (62)	I 75.8 ± 4.89 C 75.95 ± 5.65	Homes for the aged
Jung [28]	Korea	I 8 (100) C 8 (100)	I 74.3 ± 3.5 C 73.6 ± 2.4	Senior citizen center
Karahan [34]	Turkey	I 48 (43.8) C 42 (42.8)	I 71.3 ± 6.1 C 71.5 ± 4.7	–
Ku [29]	Korea	I 18 (50) C 16 (50)	I 64.7 ± 7.27 C 65.0 ± 4.77	Community-dwelling
Li [35]	China	I 40 (47.5) C 40 (42.5)	I 68.2 ± 5.5 C 69.4 ± 6.2	–
Park [30]	Korea	I 12 (25) C 12 (16.7)	I 66.5 ± 8.1 C 65.2 ± 7.9	Local community
Park [36]	Korea	I 36 (91.7) C 36 (97.2)	I 72.97 ± 2.98 C 74.11 ± 2.88	Community-dwelling
Pichierri [37]	Switzerland	I 11 (71.7) C 11 (90.9)	I 86.9 ± 5.1 C 85.6 ± 4.2	Hostel for the aged
Pluchino [41]	USA	I 12 (66.7) C 14 (64.3)	I 70.72 ± 8.46 C 76.00 ± 7.74	Community-dwelling
Pluchino [41]	USA	I 12 (66.7) C 14 (42.9)	I 70.72 ± 8.46 C 69.28 ± 6.03	Community-dwelling
Reed-Jones [31]	USA	I 15C 15	67.5 ± 5.9	Community-dwelling
Reed-Jones [31]	USA	I 15C 15	67.5 ± 5.9	Community-dwelling
Schoene [32]	Australia	I 47 (66) C 43 (67)	I 82 ± 7 C 81 ± 7	Community-dwelling
Stanmore [21]	United Kingdom	I 56 (80.4) C 50 (76)	I 77.9 ± 8.9 C 77.8 ± 10.2	Assistive living facility
Toulotte [43]	France	I 9 (55.6) C 9 (66.7)	75.09 ± 10.26	Community-dwelling
Toulotte [43]	France	I 9 (66.7) C 9 (66.7)	75.09 ± 10.26	Community-dwelling
Yang [38]	Taiwan	I 10 (90) C 10 (90)	I 68.71 (64.09–74.84) C 67.54 (62.08–76.75)	Community-dwelling
Yeşilyaprak [4]	Turkey	I 7 (42.9) C 11 (81.8)	I 70.1 ± 4.0 C73.1 ± 4.5	Rehabilitation center Nursing Home Rehabilitation Center

*I* intervention group, *C* control group

#### Sample sizes

The sample size (i.e., the sum of the intervention group and the control group) ranged from 12 [22] to 106 [21], and a total of 845 (intervention group = 428, control group = 417) participants were included in the 20 studies (Table 1).

#### Study design

All the 20 studies were randomized control trials. Twelve studies used the two-arm design, including one intervention arm and one control arm [4, 21, 23, 29, 30, 32–38]. Six studies used a three-arm design, of which four included two intervention arms and one control arm [22, 28, 39, 40], and two included one intervention arm and two control arms [31, 41]. The other two studies were a four-arm design that consisted of three intervention arms and one control arm [42, 43].

Table 2 presented the information on the interventional protocol. All the studies performed baseline and immediate post-intervention assessments of falls and balance control. Three of them consisted of assessments of falls in a longer-term follow-up period (ranging from 3-month to 1 year) [21, 32, 39]. Another three had assessments of balance control during the intervention (at 2-week, 6-week, and 3-month) [22, 31, 39]. Specifically, to measure the falls, two studies tracked and reported falls information 3-month and 6-month after the intervention [21, 32], and the other study reported the fall frequency at baseline, 6-month during the intervention, 6-month after and 12-month after the intervention [39]. One study completed the assessments of fall efficacy during the intervention, that is, at 3-month, immediately post-intervention, as well as at 6-month and 1-year follow-up [39]. The other six studies which assessed falls efficacy only had the assessments at baseline and immediately following the intervention [4, 21, 32, 37, 41, 42]. Two studies had the immediately post-intervention assessment and one additional follow-up assessment (i.e., 1-year follow-up and 4-week follow-up, respectively) to measure the balance control [33, 39], and the other 16 studies only implemented an immediately post-intervention assessment [4, 21–23, 28–32, 35, 36, 38, 40–43].

**Table 2:** Characteristics of the interventional protocol

Study	Intervention	Dosage of intervention	Types of control	Measured outcomes	Effects of intervention
Bacha [33]	Adventures game	60 min/day, 2/week, 7 week	Physical therapy exercises	BA: Mini-BESTest PIA: Mini-BESTest FUA (4-week): Mini-BESTest	Mini-BESTest→→
Bateni [22]	Balance game	3 games/day, 3/week, 4 week	Physical therapy training	BA: BBS DA (2-week): BBS PIA: BBS	BBS→
	Physical therapy training				
Bateni [22]	Balance game	3 games/d, 3/wk, 4wk	Physical therapy training	BA: BBS DA (2-week): BBS PIA: BBS	BBS→
Chen [23]	Selected Tai-Chi movements	30 min/d, 3/wk, 8wk	Traditional Tai-Chi exercise	BA: BBS, TUG, FRT PIA: BBS, TUG, FRT	BBS↑ TUG↑ FRT↑
Eggenberger [39]	Dance game	60 min/d, 2/wk, 26wk	Treadmill walking S&B training	BA: FF, FES-I, OLS DA (3-month): FES-I, OLS PIA: FF, FES-I, OLS FUA1 (6-month, 1-year): FF FUA2 (1-year): FES-I, OLS	FF→↑→ FES-I→→ OLS→→
	S&B training				
Franco [40]	Balance game	10–15 min, 2/wk, 3wk	Matter of Balance Program	BA: BBS, TB PIA: BBS, TB	BBS→ TB→
	Home exercises				
Htut [42]	Video game	30 min, 3/wk, 8wk	Physical exercises	BA: BBS, TUG, FES-I PIA: BBS, TUG, FES-I	BBS→ TUG→ FES-I↑
Jung [28]	Sport game	30 min, 2/wk, 8 wk	Lumbar stabilization exercises	BA: BBS, FRT, TUG PIA: BBS, FRT, TUG	BBS↓ FRT↓ TUG→
Karahan [34]	Sport game	30 min, 5/wk, 6 wk	Home exercise	BA: BBS, TUG PIA: BBS, TUG	BBS↑ TUG→
Ku [29]	Customized game	30 min, 3/wk, 4 wk	S & B training	BA: BBS, TUG PIA: BBS, TUG	BBS↑ TUG↑
Li [35]	Balance training	32 min, 10/month, 12 wk	One-leg standing exercise	BA: TUG, SL PIA: TUG, SL	TUG↑ SL↑
Park [30]	Balance game/program	30 min, 3/wk, 8 wk	Ball exercise	BA: TUG, SL, SS PIA: TUG, SL, SS	TUG↑ SL↑ SS→
Park [36]	Kayak program	50 min, 2/wk, 6 wk	Conventional exercise	BA: PC standing PIA: PC standing	PC standing↑ PC sitting↑
	Conventional exercise				
Pichierri [37]	Resistance training Balance training	50–55 min, 2/wk, 12 wk	Resistance and balance training	BA: FES-I PIA: FES-I	FES-I→
	Dance game				
Pluchino [41]	Balance game	60 min, 2/wk, 8wk	Standardized balance exercise program	BA: FES, TUG, OLS, FRT, TB, SL PIA: FES, TUG, OLS, FRT, TB, SL	FES→ TUG→ OLS→ FRT→ TB→ SL→
Pluchino [41]	Balance game	60 min, 2/wk, 8 wk	Tai Chi	BA: FES, TUG, OLS, FRT, TB, SL PIA: FES, TUG, OLS, FRT, TB, SL	FES→ TUG→ OLS→ FRT→ TB→ SL→
Reed-Jones [31]	Agility training Visual training	90 min, 2/wk, 12 wk	ACSM exercises for elderly	BA: FRT, TUG DA (6-week): FRT, TUG PIA: FRT, TUG	FRT→ TUG→
	ACSM exercises for elderly				
Reed-Jones [31]	Agility training Visual training	90 min, 2/wk, 12 wk	ACSM exercises for elderly Agility training	BA: FRT, TUG DA (6-week): FRT, TUG PIA: FRT, TUG	FRT→ TUG→
	ACSM exercises for elderly				
Schoene [32]	Stepping game	20 min, 3/wk, 16 wk	Home exercise	BA: Icon-FES, TUG PIA: Icon-FES, TUG FUA (6-month): number of fallers	Number of fallers→ Icon-FES↑ TUG↑
Stanmore [21]	Exergame	30 min, 3/wk, 12 wk	S & B exercises	BA: BBS, FES-I, TUG PIA: BBS, FES-I, TUG FUA (3-month): FI (includes the number of falls and fallers)	BBS↑ FES-I↑ TUG→ FI↑
	S & B exercise				
Toulotte [43]	Sport game	60 min, 1/wk, 20 wk	Adapted physical training	BA: UT, TB, COG PIA: UT, TB, COG	UT↓ TB↓ COG↑
Toulotte [43]	Adapted physical activities Sport game	60 min, 1/wk, 20 wk	Adapted physical training	BA: UT, TB, COG PIA: UT, TB, COG	UT↓ TB↓ COG↑
Yang [38]	Exergame	45 min, 2/wk, 5 wk	Balance training	BA: TUG, FRT, OLS PIA: TUG, FRT, OLS	TUG→ FRT↑ OLS→
Yeşilyaprak [4]	Balance exercise	35–45 min, 3/wk, 6 wk	Balance exercises	BA: BBS, TUG, OLS, FES-I PIA: BBS, TUG, OLS, FES-I	BBS→ TUG→ OLS→ FES-I→

*ACSM* The American College of Sports Medicine, *S & B* strength and balance, *min* minute(s), *d* day(s), *wk* week(s), *BA* baseline assessment, *DA* during assessment, *PIA* post-intervention assessment, *FUA* follow-up assessment, *FES-I* The Falls Efficacy Scale International, *FES* Falls Efficacy Scale, *BBS* Berg Balance Scale, *TUG* timed up and go, *OLS* one-leg stance, *FF* fall frequency, *FI* fall incidence, *FRT* functional reach test, *TB* Tinetti balance, *SL* sway length, *SS* sway speed

#### Interventions

Based on the motor learning principles [27], the exergame interventions can be categorized into five types (Table 3). Twelve studies implemented the EI-only [4, 23, 28–30, 32–35, 38, 41, 42]; six studies implemented the combined intervention [21, 31, 36, 37, 39, 40]; and two studies included both the EI-only and combined interventions [22, 43].

**Table 3:** Classification of exergames according to the motor learning principles

Principles of motor learning	To enhance	Intervention model	Intervention system
Learning occurs through repetitive, varied practice of meaningful tasks	Balance (proprioception), postural control, strength, motor coordination, and cognition	Balance game/exercise [22, 30, 31, 35, 40], sport game (such as football, tennis, skiing game and simulations) [28, 34, 38], the kayak program [36], selected Tai Chi movements [23], customized tasks [29]	Balance-A [35], Microsoft Kinect [34, 38], AR (Microsoft Kinect) [23, 29], Nintendo Wii [28, 40], Nintendo Wii with balance board [22, 31], VR [30, 36]
Learning occurs when task difficulty is progressively increased according to the user's ability	Aerobic endurance, balance, postural control, strength, motor coordination, and cognition	Balance game/exercise [4, 41], dance game [37, 39], exergame [42], tailored exergame program [21], steeping game [32], selected Tai Chi movements [23], and sport game [43]	Microsoft Kinect [21], AR with Microsoft Kinect [23], the interact training system [32], Nintendo Wii [41, 43], VR (X-box 360) [42], VR with dance pad/platform [4, 37, 39]
Learning occurs when the individual is motivated to improve	Balance control, motor coordination, and cognition	Exergame [42], stepping game [32]	VR (X-box 360) [42], the interact training system [32]
Sensory feedback that is related to the task is necessary for learning	Balance control	Balance exercise [4]	VR (BTS NIRVANA) [4]
Learning occurs when an individual receives positive feedback about task performance and task accomplishment	Balance, stepping ability, and cognition	Stepping game [32], Adventure game [33]	The interact training system [32], VR (Microsoft Kinect) [33]

*VR* virtual reality

The intervention duration was from 3 to 26-week, with a frequency between one and five times per week. The length of each intervention session ranged from 10 to 90 min (Table 2). In 12 studies, exergame interventions were supervised by physical therapists, therapist assistants, and investigators [4, 21, 22, 29, 33, 34, 37–40, 42, 43]; the intervention was completed without any supervision in two studies [32, 41]; the other six studies did not report this information [23, 28, 30, 31, 35, 36].

#### The types of the control intervention

The following traditional physical interventions of the control group were used in the studies:

- Physical therapy exercises to improve strength, flexibility, endurance, postural control, and balance [22, 33].
- Physical training exercises that challenge various physical abilities to improve endurance, strength, balance, flexibility, and agility [21, 29, 31, 32, 34, 36–38, 40, 42, 43].
- Balance exercises to improve balance [4, 30, 35, 41].
- Tai Chi to improve lower-body strength, postural control, and balance [23, 41].
- Treadmill walking to improve aerobic endurance [39].
- Lumbar stabilization exercises improve truck muscle strength and static balance [28].

#### Study outcomes

The meta-analysis quantitatively assessed the effects of EI on falls and balance control (Table 2). The outcomes used to assess falls were the number of fallers (participants who experienced falls), number of falls, and fall efficacy. Specifically, two studies reported the number of fallers and falls during the follow-up period [21, 32], while Eggenberger et al. [39] only reported the incidence of falls but did not respond to the inquiry of our team for more specific information. The fall efficacy was assessed by Falls Efficacy Scale-International (FES-I) in five studies [4, 37, 39, 41, 42], by Iconographical Fall-Efficacy Scale (Icon-FES) in one study [32], and by short FES-I in one study [21]. Across all the studies, thirteen only assessed the effects of interventions on balance control [22, 23, 28–31, 33–36, 38, 40, 43]; one only assessed the effects of EI on falls [37], and the other six studies assessed the effects on both falls and balance [4, 21, 32, 39, 41, 42].

To assess the effects of EI on balance control, we first categorized the outcomes into those related to static balance and those related to dynamic balance. Specifically, the Berg Balance Scale (BBS), Tinetti balance Assessment [4, 21–23, 28, 29, 34, 40–43], one-leg stance performance (OLS) [4, 38, 39, 41, 43], functional reach test (FRT) [23, 28, 31, 38, 41], and the postural sway path length and mean sway speed of the center of pressure movement were outcomes of static balance [30, 35, 36, 41, 43]. Dynamic balance is assessed by the completion time of the timed-up-and-go test (TUG) [4, 21, 23, 28–32, 34, 35, 38, 41, 42]. Twenty studies assessed the immediate effects of the EI on balance control, and only two studies assessed the longer-term effects of EI on balance control, and the follow-up assessments were completed at 4-week and 1 year after the intervention [33, 39].

#### The effects of exergame interventions on falls and balance control

Table 2 showed the effects of EI compared to PE on falls and balance control. As compared to the control, one study reported that the intervention induced a greater reduction of fall incidence during the 3-month follow-up [21], and the other two studies [32, 39] reported no between-group difference in fall frequency during the following 6-month follow-ups. Three studies showed the intervention induced a greater reduction in fall efficacy as compared to the control [21, 32, 42], and the other four studies showed no between-group difference [4, 37, 39, 41].

For static balance assessed by BBS and Tinetti balance Assessment, four studies reported that the intervention group had greater improvement [21, 23, 29, 34], five studies reported no significant difference between groups [4, 22, 40–42], and three studies showed that the control group had greater improvement [22, 28, 43]. For OLS, one study [43] reported the control induced greater improvement, while the other studies reported no between-group difference. For FRT, two studies showed the intervention produced greater improvement [23, 38], one study showed the opposite result [28], and the other studies reported no between-group difference. For postural control assessed by sway path length of the center of pressure, one study showed no between-group difference [41], and others showed that the intervention group led to a greater improvement. There was one study reporting a greater reduction in sway speed of the center of pressure after the intervention [36], and two studies showed no between-group difference [30, 41]. For TUG, compared to the control, the intervention showed greater improvement in dynamic balance measured by TUG tests in five studies [23, 29, 30, 32, 35], and others reported no between-group difference.

Regarding the carry-over effect of interventions on balance control, Bacha et al. [33] reported that both conventional physical therapy and Kinect adventures training significantly improved participants’ balance control at post-intervention and was maintained at 4-week follow-up assessment, which was in line with Eggenberger et al. [39].

### Quality assessment

**Fig. 2:**
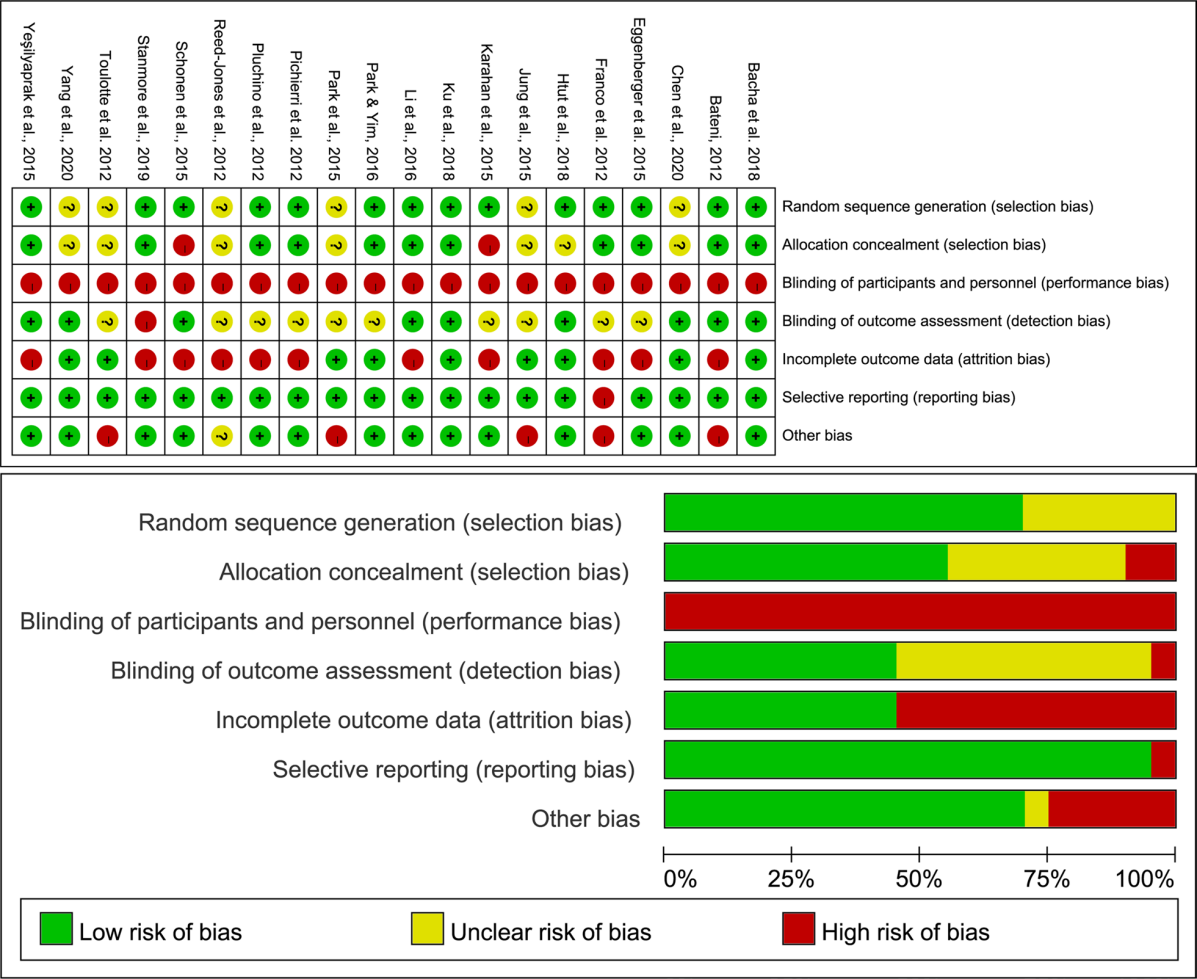
Analysis of the risk of bias in accordance with the Cochrane Collaboration Guidelines

The results of the methodological quality assessment are presented in Fig. 2. One study implemented double-blinded trial protocol [22]; nine studies were single-blinded [4, 23, 29, 32, 33, 35, 38, 39, 42]; three studies were open-labeled [21, 37, 40], and seven studies did not report the related information [28, 30, 31, 34, 36, 41, 43].

### Meta-analysis results

Due to the lack of enough research providing the number of fallers and falls (n = 2) [21, 32], we did not perform a meta-analysis on the two outcomes.

#### Effects of exergame on fall efficacy

**Fig. 3:**
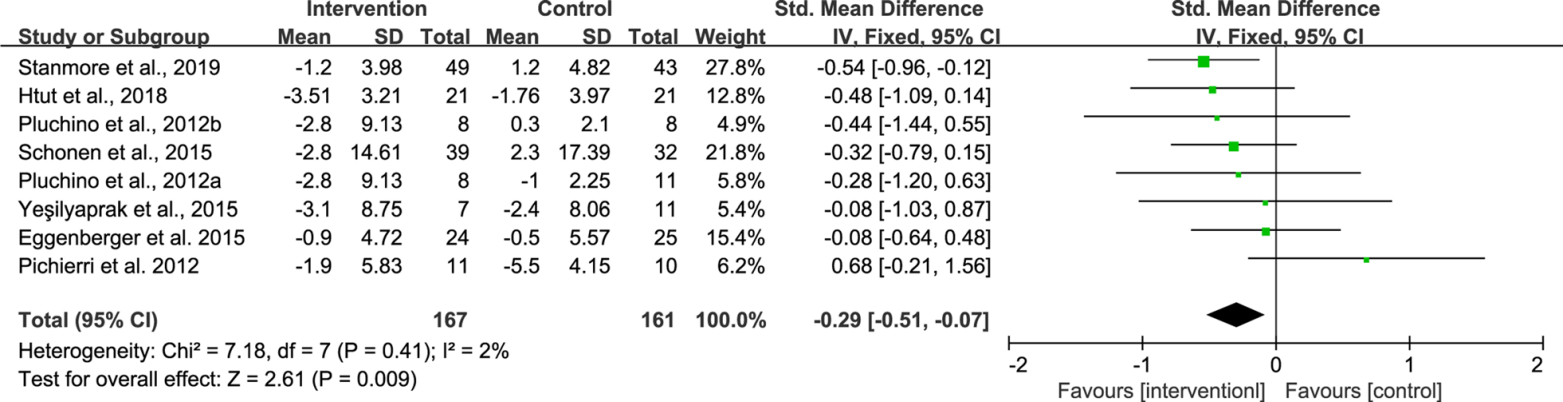
Result of meta-analysis for fall efficacy

Seven studies examined the effects of exergame intervention on fall efficacy [4, 21, 32, 37, 39, 41, 42]. As compared to the control, the intervention was revealed to induce greater reduction in fall efficacy (SMD = − 0.29, 95% CI − 0.51 to − 0.07, *P* = 0.009, *I*^2^ = 2%) (Fig. 3). Sensitivity analysis showed one study had a much larger effect size than the other studies [37], and the *I*^2^ dropped to 0% after removing this study, although the pooled effect size was not changed (SMD = − 0.36, 95% CI − 0.58 to − 0.13, *P* = 0.002).

#### Effects of exergame on static balance

Eleven studies reported the effects of exergame intervention on BBS [4, 21–23, 28, 29, 34, 40, 42] and Tinetti Balance assessment [40, 41, 43]. Five studies reported results for OLS performance with eyes open and eyes closed [4, 38, 39, 41, 43]. Five studies reported intervention effects on FRT distance [23, 28, 31, 38, 41]. Four studies reported on sway path length measured in standing position [30, 35, 41, 43]. Three studies evaluated the effects of exergame intervention on sway speed [30, 36, 41].

**Fig. 4:**
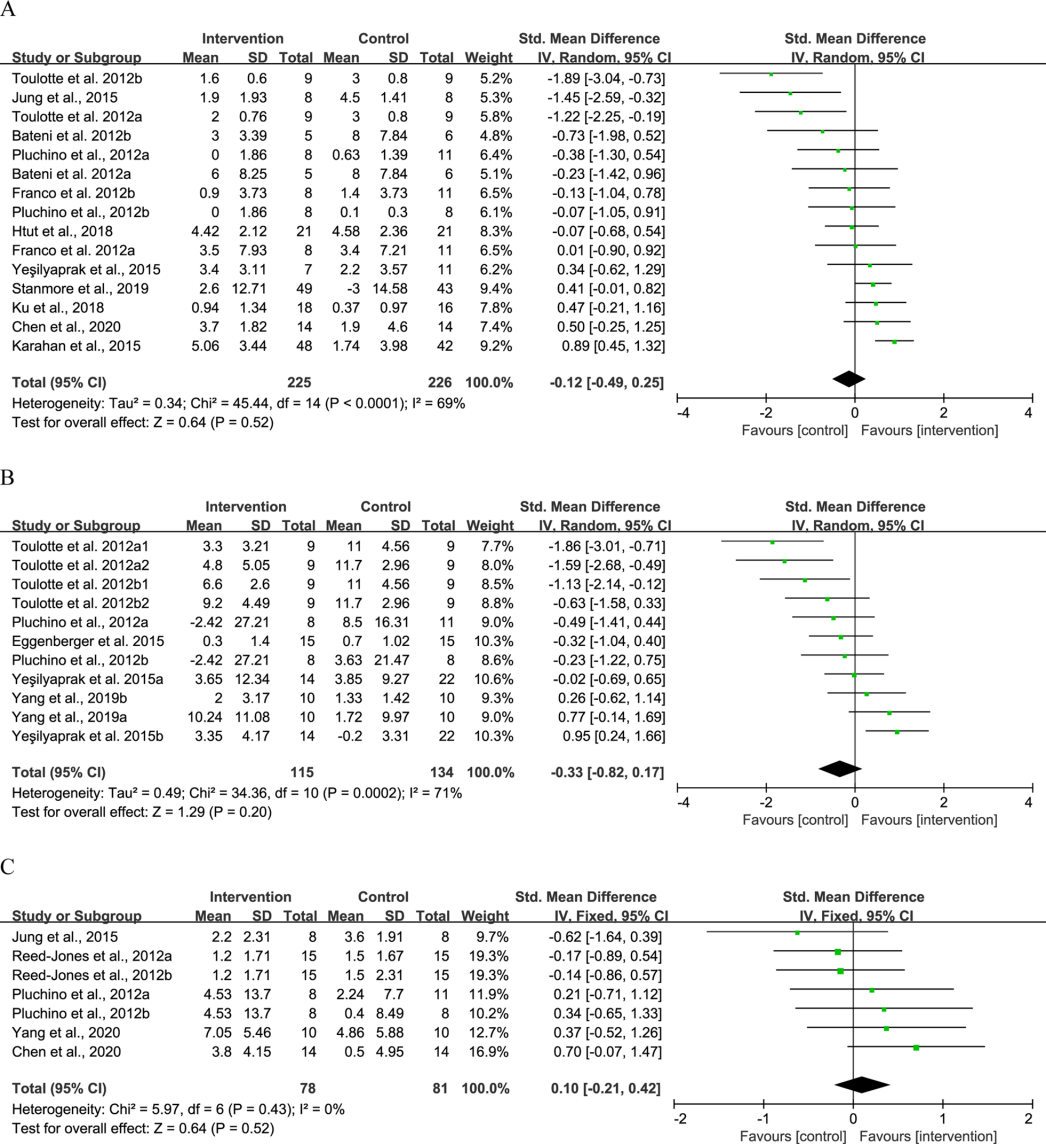
Result of meta-analysis for static balance. **A** BBS, **B** OLS, and **C** FRT

**Fig. 5:**
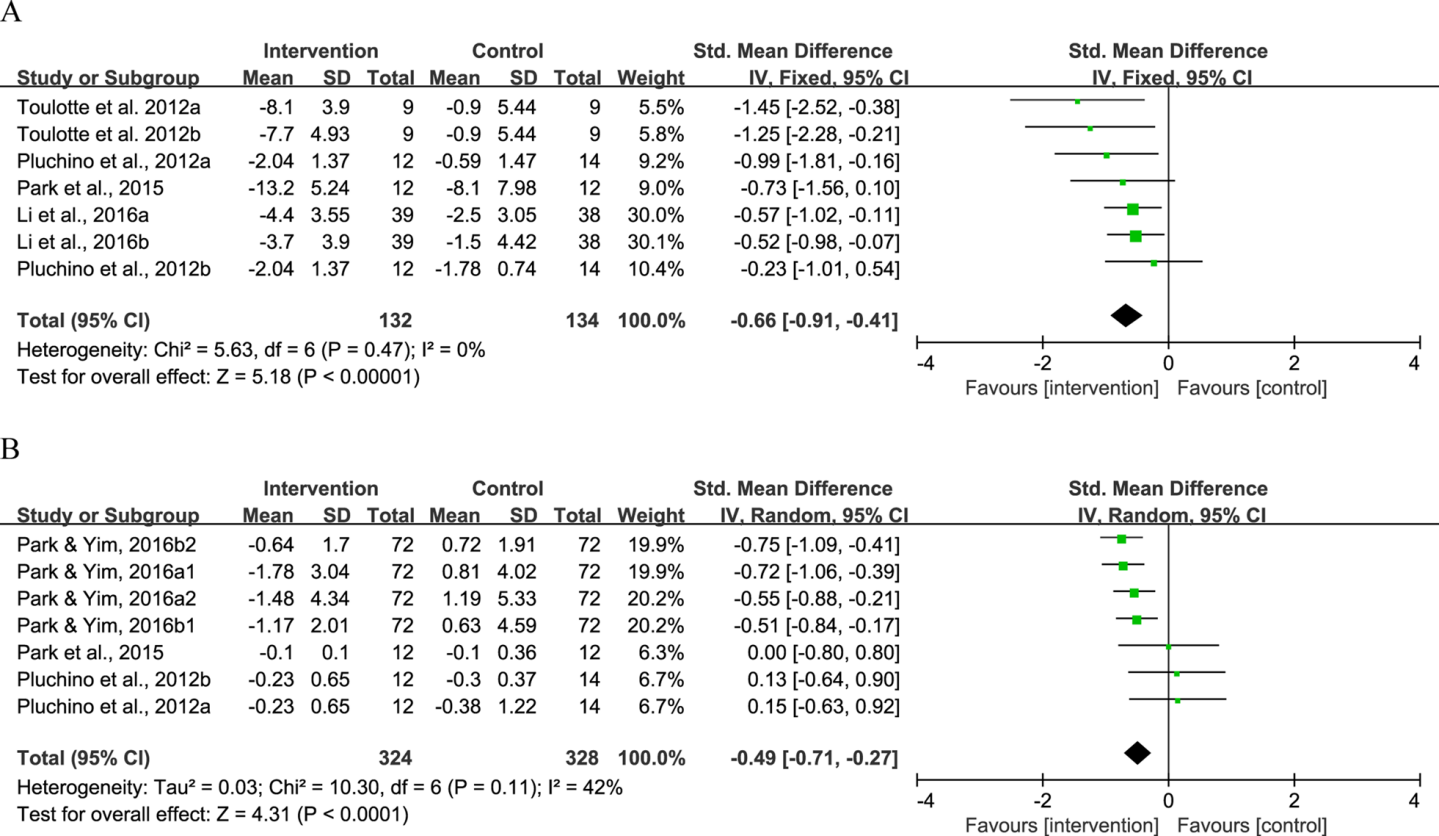
Result of meta-analysis for postural control. **A** Sway path length and **B** sway speed. Sway length test was measured by force platform in standing position with eyes open [30, 35, 41] and eyes closed [35]. Sway speed was measured with eyes open in standing position for Park et al. [30] and Pluchino et al. [41], while Park and Yim [36] reported data of both standing and sitting positions with eyes open and closed

No significant difference was observed in BBS (SMD = − 0.12, 95% CI − 0.49 to 0.25, *P* = 0.52, *I*^2^ = 69%), OLS (SMD = − 0.33, 95% CI − 0.82 to 0.17, *P* = 0.20, *I*^2^ = 71%), and FRT (SMD = 0.10, 95% CI − 0.21 to 0.42, *P* = 0.52, *I*^2^ = 0%) between the intervention and control groups (Fig. 4). As compared to the control, the intervention could induce greater reduction in sway path length (SMD = − 0.66, 95% CI − 0.91 to − 0.41, *P* < 0.001, *I*^2^ = 0%) (Fig. 5A) and sway speed (SMD = − 0.49, 95% CI − 0.71 to − 0.27, *P* < 0.001, *I*^2^ = 42%) (Fig. 5B).

A sensitivity analysis was conducted to explore the possible impact of bias. For BBS, three studies that having a much larger effect size than the other studies were removed from the meta-analysis [28, 34, 43], the *I*^2^ dropped from 69 to 0% without any change to the pooled effect size (SMD = − 0.16, 95% CI − 0.06 to 0.39, *P* = 0.16, *I*^2^ = 0%). For OLS, we removed three studies that had larger effect size than the other studies [4, 38, 43], and the *I*^2^ dropped from 71 to 0% with no change occurred to the pooled effect size (SMD = − 0.30, 95% CI − 0.62 to 0.02, *P* = 0.07, *I*^2^ = 0%). For sway speed, sensitivity analysis showed one study had a much larger effect size than the other studies [36]. After removing the study, the *I*^2^ dropped to 0% and the pooled effect size remained unchanged (SMD = 0.09, 95% CI − 0.36 to 0.55, *P* = 0.68, *I*^2^ = 0%).

#### Effects of exergame on dynamic balance

**Fig. 6:**
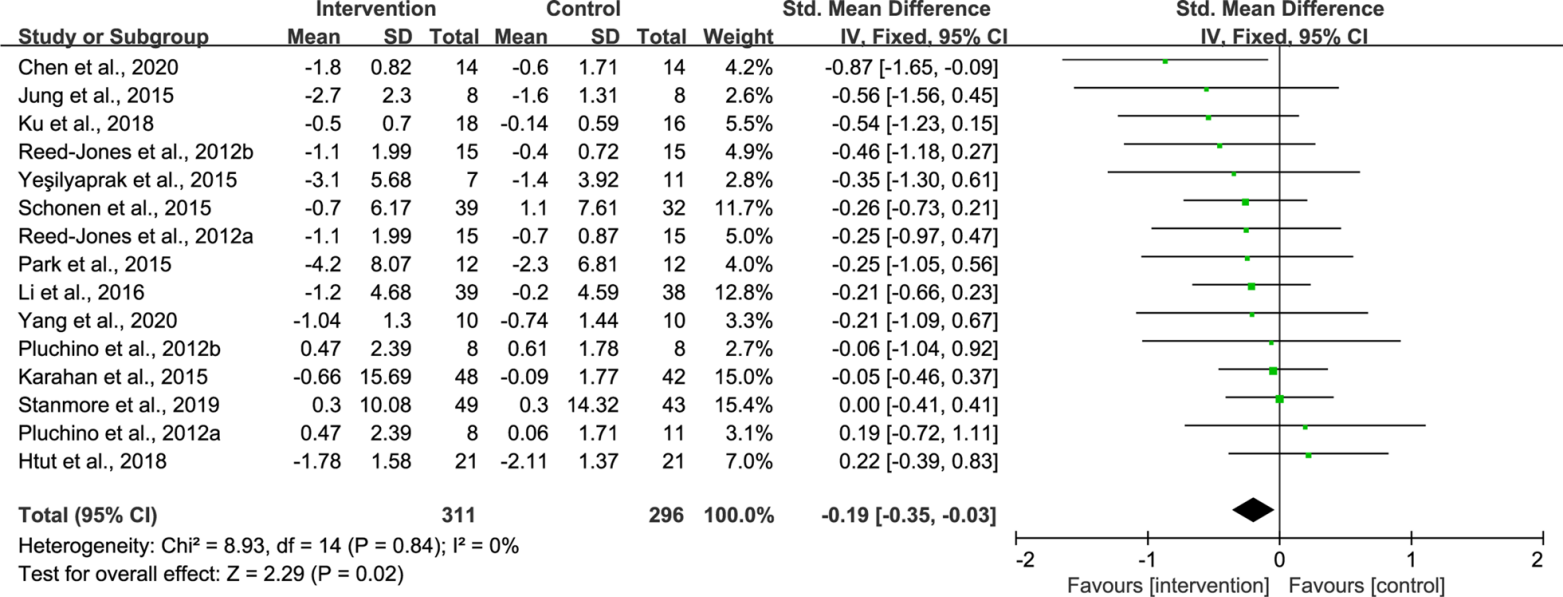
Result of meta-analysis for TUG. The TUG test in Schoene et al. [32] was measured with a concurrent secondary task

Thirteen studies reported intervention effects on dynamic balance by means of TUG tests [4, 21, 23, 28–32, 34, 35, 38, 41, 42]. As compared to the control, the intervention could induce greater improvement in dynamic balance (SMD = − 0.19, 95% CI − 0.35 to − 0.03, *P* = 0.02, *I*^2^ = 0%) (Fig. 6).

### Subgroup analysis

#### Different types of exergames

To further assess the effects of different exergames on falls prevention and balance control, which were designed by different principles of motor learning, subgroup analysis results were presented in Additional file 1: Figure S1. Due to the lack of studies in some categories, we performed the analysis on fall efficacy, BBS and TUG. It was observed that: (1) fall efficacy was significantly reduced by exergames designed by the principle of progressively increasing the difficult of task (SMD = − 0.29, 95% CI − 0.51 to − 0.07, *P* = 0.009, *I*^2^ = 2%), but not by exergames designed by the other two principles: providing sensor feedback that is related to the task and presenting positive feedback of task performance (SMD = − 0.38, 95% CI − 0.75 to − 0.00, *P* = 0.05, *I*^2^ = 0%; SMD = − 0.32, 95% CI − 0.79 to 0.15, *P* = 0.19); (2) TUG performance was improved by exergames designed by the principle of repetitive and varied practice of meaningful tasks (SMD = − 0.25, 95% CI − 0.47 to − 0.02, *P* = 0.03, *I*^2^ = 0%), but not by exergames designed by the following four different principles: progressively increasing the difficult of task, motiving participants’ performance by presenting the task performance, providing sensor feedback related to the task, and presenting positive feedback of task performance (SMD = − 0.12, 95% CI − 0.36 to 0.11, *P* = 0.31, *I*^2^ = 1%; SMD = − 0.08, 95% CI − 0.45 to 0.29, *P* = 0.67, *I*^2^ = 33%; SMD = − 0.35, 95% CI − 1.30 to 0.61, *P* = 0.48; SMD = − 0.26, 95% CI − 0.73 to 0.21, *P* = 0.28).

#### Different types of interventions

To assess the effects of the EI-only and the combined intervention on fall prevention and balance control, subgroup analysis results were presented in Additional file 2: Figure S2. It was observed that: (1) fall efficacy was significantly reduced by the EI-only (SMD = − 0.34, 95% CI − 0.65 to − 0.03, *P* = 0.03, *I*^2^ = 0%), but not by the combined intervention (SMD = − 0.24, 95% CI − 0.56 to 0.07, *P* = 0.13, *I*^2^ = 69%), with no significant difference between the two subgroups (*P* = 0.67); (2) OLS performance was significantly improved by the combined intervention (SMD = − 0.60, 95% CI − 1.10 to − 0.10, *P* = 0.02, *I*^2^ = 0%), but not by the EI-only (SMD = − 0.21, 95% CI − 0.86 to 0.43, *P* = 0.52, *I*^2^ = 76%), with no significant difference between the two subgroups (*P* = 0.35); (3) the reduction of sway length was significant in both the EI-only and the combined intervention (SMD = − 0.62, 95% CI − 0.88 to − 0.37, *P* < 0.001; SMD = − 1.25, 95% CI − 2.28 to − 0.21, *P* = 0.02, *I*^2^ = 0%), with no significant subgroup difference (*P* = 0.25); (4) sway speed was significantly reduced by the combined intervention (SMD = − 0.63, 95% CI − 0.80 to − 0.46, *P* < 0.001, *I*^2^ = 0%), but not by the EI-only (SMD = 0.09, 95% CI − 0.36 to 0.55, *P* = 0.68, *I*^2^ = 0%), and the combined intervention was significantly better than the EI-only (*P* = 0.003); (5) TUG performance was significantly improved by the EI-only (SMD = − 0.20, 95% CI − 0.39 to − 0.02, *P* = 0.03, *I*^2^ = 0%), but not by the combined intervention (SMD = − 0.14, 95% CI − 0.46 to 0.18, *P* = 0.40, *I*^2^ = 0%), with no significant difference between the two subgroups (*P* = 0.73).

#### The dose–response effect of exergame interventions

The subgroup analyses on program period (i.e., 1 to 8-week and more than 8) and weekly intervention duration (i.e., 1 to 89 min, 90 to 119 min, and more than 119 min) were conducted to assess the dose–response effect of the EI (see Additional file 3: Figure S3 and Additional file 4: Figure S4).

For subgroups of program period, it was observed that: (1) fall efficacy was significantly reduced for more than 8-week (SMD = − 0.27, 95% CI − 0.53 to − 0.01, *P* < 0.05, *I*^2^ = 0%), but not for 1 to 8-week (SMD = − 0.36, 95% CI − 0.77 to 0.05, *P* = 0.09, *I*^2^ = 0%), with no significant subgroup difference (*P* = 0.71); (2) sway path length was significantly reduced for 1 to 8-week and more than 8 (SMD = − 0.63, 95% CI − 1.10 to − 0.16, *P* = 0.008, *I*^2^ = 0%; SMD = − 0.67, 95% CI − 0.97 to − 0.38, *P* < 0.001, *I*^2^ = 22%), with no significant subgroup difference (*P* = 0.89). Notably, PT induced greater improvement in OLS than EI for more than 8-week (SMD = − 1.01, 95% CI − 1.59 to − 0.43, *P* < 0.001, *I*^2^ = 44%).

For subgroups of weekly intervention duration, it was observed that: (1) fall efficacy was significantly reduced by 90–119 min (SMD = − 0.36, 95% CI − 0.68 to − 0.04, *P* = 0.03, *I*^2^ = 67%), but not by less than 90 min (SMD = − 0.32, 95% CI − 0.79 to 0.15, *P* = 0.19, *I*^2^ = 0%) and more than 119 min (SMD = − 0.17, 95% CI − 0.56 to 0.22, *P* = 0.39, *I*^2^ = 0%); (2) BBS was significantly improved by 90–119 min (SMD = 0.33, 95% CI 0.04 to 0.61, *P* = 0.02, *I*^2^ = 0%), but not by more than 119 min (SMD = 0.28, 95% CI − 0.36 to 0.93, *P* = 0.39, *I*^2^ = 62%). Notably, traditional physical training induced greater improvement in BBS and OLS than exergame intervention by less than 90 min (SMD = − 0.63, 95% CI − 1.13 to − 0.14, *P* = 0.01, *I*^2^ = 51%; SMD = − 1.24, 95% CI − 1.77 to − 0.70, *P* < 0.001, *I*^2^ = 4%); (3) sway length was significantly reduced by less than 90 min (SMD = − 0.67, 95% CI − 0.97 to − 0.38, *P* < 0.001, *I*^2^ = 22%) and more than 119 min (SMD = − 0.59, 95% CI − 1.15 to − 0.02, *P* = 0.04, *I*^2^ = 41%), but not by 90 to 119 min (SMD = − 0.73, 95% CI − 1.56 to 0.10, *P* = 0.09); (4) sway speed was significantly reduced by 90–119 min (SMD = − 0.60, 95% CI − 0.77 to − 0.44, *P* < 0.001, *I*^2^ = 0%), but not by more than 120 min (SMD = 0.14, 95% CI − 0.41 to 0.68, *P* = 0.62, *I*^2^ = 0%).

### Publication bias

**Fig. 7:**
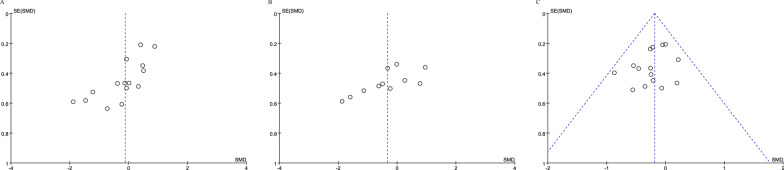
Funnel plot for publication bias assessment. **A** BBS, **B** OLS, and **C** TUG

The funnel plot did not show a clear funnel shape in BBS and OLS (Fig. 7A, B). This may be explained by the following two reasons: first, some small studies with negative results did not favor exergame intervention; second, the intervention effects of different interventions seemed to be distinct, which led to the existence of heterogeneity and made the funnel chart asymmetric. The funnel plot for TUG showed no publication bias (Fig. 7C). The number of studies on the left and right of the dashed standardized mean difference line is equally distributed.

## Discussion

This systematic review and meta-analysis suggests that exergame intervention is a promising strategy to improve the performance of balance control and reduces falls in relatively healthy older adults. The results of meta-analyses indicated that the EI induced a greater improvement in fall efficacy, postural control, and dynamic balance as compared to traditional training. Still, future studies with rigorous study design, larger sample size, and longer-term follow-up assessments are needed to further examine the effectiveness of different types of EI, as well as to characterize the dose–response effect of the EI and the time length such effects can sustain. With this knowledge in hand, it will thus ultimately help optimize the design of exergame-based intervention in future rehabilitation programs of balance, mobility, and fall prevention for older adult population.

The results of our work revealed that compared to traditional physical training, EI induced greater reduction in falls. One potential reason is that traditional physical exercise mainly focuses on physical function, while EI concentrate on both physical function and other functions that are important to prevent the incidence of falls, such as balance, mobility, cognitive function, and mood [19, 44–49]. Therefore, implementing exergames that simultaneously challenge multiple functional components (e.g., cognition, balance, strength, and motor coordination) might be a helpful strategy to prevent falls in healthy older people. Moreover, the subgroup-analysis revealed that the exergames that gradually increase in difficulty could induce a significant reduction in fall efficacy. However, due to the limited number of included studies in some subgroups (e.g., only one study implemented EI designed based on the principle of positive feedback [32]; Additional file 1: Figure S1), this finding should be treated with caution. Meanwhile, EI with 90–119 min for more than 8-week induced the largest reduction in fall incidence. However, to date, most studies focused on the immediate effects of EI (e.g., within 7 days after the intervention) [41], and the longer-term effects were still unclear. Moreover, several studies (three out of seven) consisted of a small sample size of participants (fewer than 30). Therefore, futures studies with a larger sample and longer-term follow-up assessment (e.g., a 1-year follow-up to track the falls according to the guidelines of the Prevention of Falls Network Europe Consensus [50]) are thus needed to confirm the findings in these pilot studies and to examine the longer-term effects of EI on fall risk.

The meta-analyses also demonstrated that compared to traditional physical training, EI induced greater improvement in the performance of balance control. This is consistent with the observations in another meta-analysis [51]. Such improvements induced by EI may arise from the augmentation in the capacity to integrate sensory information (i.e., the visual and somatosensory inputs [52]), in vestibular functions (e.g., gaze stability during head movements) [53] and in the improved cognitive function (e.g., attention) [54].

It should be noted that the results of the meta-analysis showed that the significant improvements with small to moderate magnitude were observed only in postural control and dynamic balance, but not in static balance performance (i.e., BBS, OLS, and FRT). This may be due to the potential ceiling effect [55], that is, the studies included in this work focused on healthy older adults so that the performance of their static balance was rather excellent even before the intervention. For example, the average value of the functional reaching test results at baseline (26.92 ± 8.02 cm) of the included studies is greater than the reported normative level of older adult population (26.6 cm) [56]. Therefore, very limited room is available for improving more. The effects of EI on people with limited functionalities (e.g., slowed gait) are worthwhile to be examined.

Notably, the subgroup analysis indicated that the repeated implementation of diverse and meaningful exergames can effectively improve the balance control of healthy elderly people. Nonetheless, the effects of exergames designed based on the other principles to improve balance control and prevent falls are still not well-characterized. The subgroup analysis examining the relationship between the intervention length and effects showed that the EI of 90–119 min per week induced the largest improvement in balance control; but the number of intervention sessions or the intervention duration that can induce the largest improvement is still uncertain because inconsistent effects of exergame intervention on balance control (i.e., OLS and sway length) were observed. On the other hand, when participants completed the intervention fewer than 90 min per week, it was observed that the traditional physical training induced greater improvement in static balance as compared to EI. One possible reason for this finding was that exergame-based exercise included multicomponent cognitive-physical stimulations, which may require a longer intervention duration to achieve the expected improvements. Therefore, this may suggest that when the time available for intervention is limited, healthy older adults may benefit more from participating in the traditional physical training intervention.

The effects of the EI-only and the combined intervention are also compared in this study. It is observed that as compared to EI-only, the combined intervention induced greater improvements in balance control. One explanation of this finding may be that the combination of exergame intervention and physical training not only enhanced the physical function of older adults (e.g., strength), but also reinforced their cognitive function (e.g., working memory, attention, and information processing speed) [54]. All these functionalities are closely related to the control of balance [52], so that such combined intervention can bring more benefits to balance control in older adults. Unfortunately, until now, only few studies compared the effects of these two types of intervention on fall prevention, and more studies are needed to examine if the combined intervention reduce more falls in older adults.

Several limitations should be noted in the methodology of existed publications. First, only 20 publications reporting the results of RCTs were eligible and included in this analysis, which may potentially limit the power of the evidence. This suggests that more work is needed in the field to further examine and confirm the current findings. Second, the existence of publication bias resulted in heterogeneity (e.g., the variance in the protocol of intervention) between included studies, which reduced the quality of evidence. One important variance contributing to the inconsistent results is that some studies used customized exergame, that is, the intervention was designed based upon the study aims and well-suited for the study population, while other studies simply used the commercialized exergame (e.g., Wii Fit balance games: Ski Slalom, Ski Jump and Table Tilt) without the customization. Studies are thus needed to examine if the customization of exergame can help augment the benefits. Third, several important aspects of EI have not been well characterized, including the longer-term effects, the optimal duration or number of sessions that induces the largest improvements, and the comparison of effects between EI-only and combined intervention.

## Conclusion

Our systematic review and meta-analysis demonstrate that EI, especially the combination of EI and PT, is a novel and promising strategy that can help improve balance control and reduce falls in older adults. The EI with a longer program duration (more than 8-week) and 90–119 min/week is recommended to induce greater benefits. Implementation of diverse and meaningful exergames and exergames that gradually increase in difficulty could improve balance control and prevent falls in healthy older adults, but the finding should be further examined and confirmed. Future studies with rigorous design, larger sample size, longer-term follow-up assessments are thus needed, of which the findings can help optimize the design of EI and its benefits for balance and fall prevention in the older adult population.

## Supplementary Information

**Additional file 1: Figure S1.** Result of subgroup meta-analysis by principles of motor learning. (A) Fall efficacy, (B) BBS, and (C) TUG.

**Additional file 2: Figure S2.** Result of subgroup meta-analysis by intervention type. (A) Fall efficacy, (B) BBS, (C) OLS, (D) FRT, (E) sway length, (F) sway speed, and (G) TUG.

**Additional file 3: Figure S3.** Result of subgroup meta-analysis by program period. (A) Fall efficacy, (B) BBS, (C) OLS, (D) FRT, (E) sway length, and (F) TUG.

**Additional file 4: Figure S4.** Result of subgroup meta-analysis by weekly intervention duration. (A) Fall efficacy, (B) BBS, (C) OLS, (D) FRT, (E) sway length, (F) sway speed, and (G) TUG.

## Data Availability

The datasets supporting the conclusions of this article are included within the article.

## Abbreviations

BA: Baseline assessment
BBS: Berg Balance Scale
CI: Confidence interval
DA: During assessment
EI: Exergame intervention
FUA: Follow-up assessment
FRT: Functional reach test
MD: Mean difference
OLS: One-leg stance performance
PIA: Post-intervention assessment
PRISMA: Preferred Reporting Items for Systematic Reviews and Meta-Analyses
PT: Physical training
MeSH: Medical Subject Headings
RCT: Randomized controlled trial
S & B: Strength and balance
SD: Standard deviation
SE: Standard error
SMD: Standardized mean difference
TUG: Timed-up-and-go test
VR: Virtual reality
