# Supplementary material for: Comparison between the effects of exergame intervention and traditional physical training on improving balance and fall prevention in healthy older adults: a systematic review and meta-analysis
Source: J Neuroeng Rehabil. 2021 Nov 24;18:164. doi: 10.1186/s12984-021-00917-0 (PMC8611920; doi:10.1186/s12984-021-00917-0)
Supplement: Supplementary file 1 — Additional file 1: Figure S1. Result of subgroup meta-analysis by principles of motor learning. (A) Fall efficacy, (B) BBS, and (C) TUG. [file 12984_2021_917_MOESM1_ESM.docx]

A


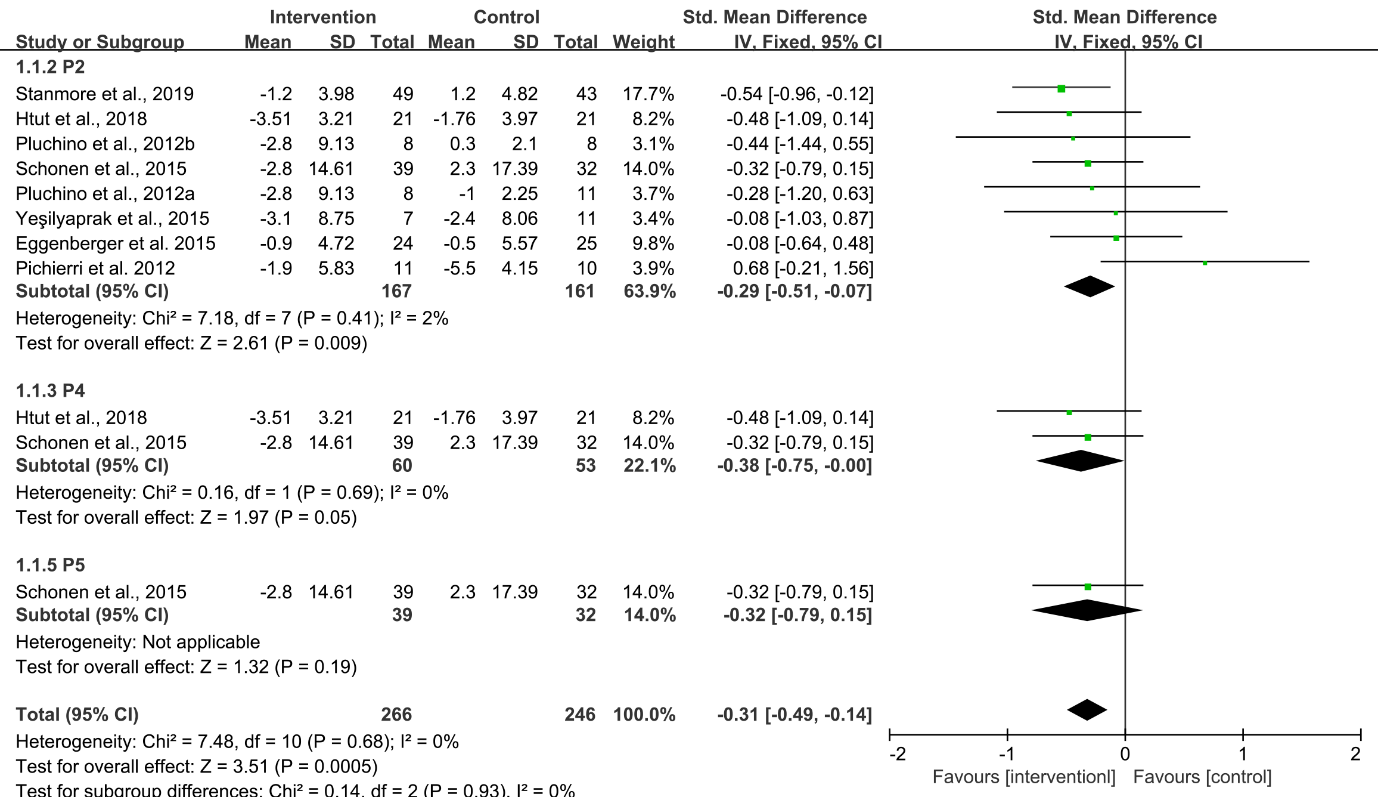


B


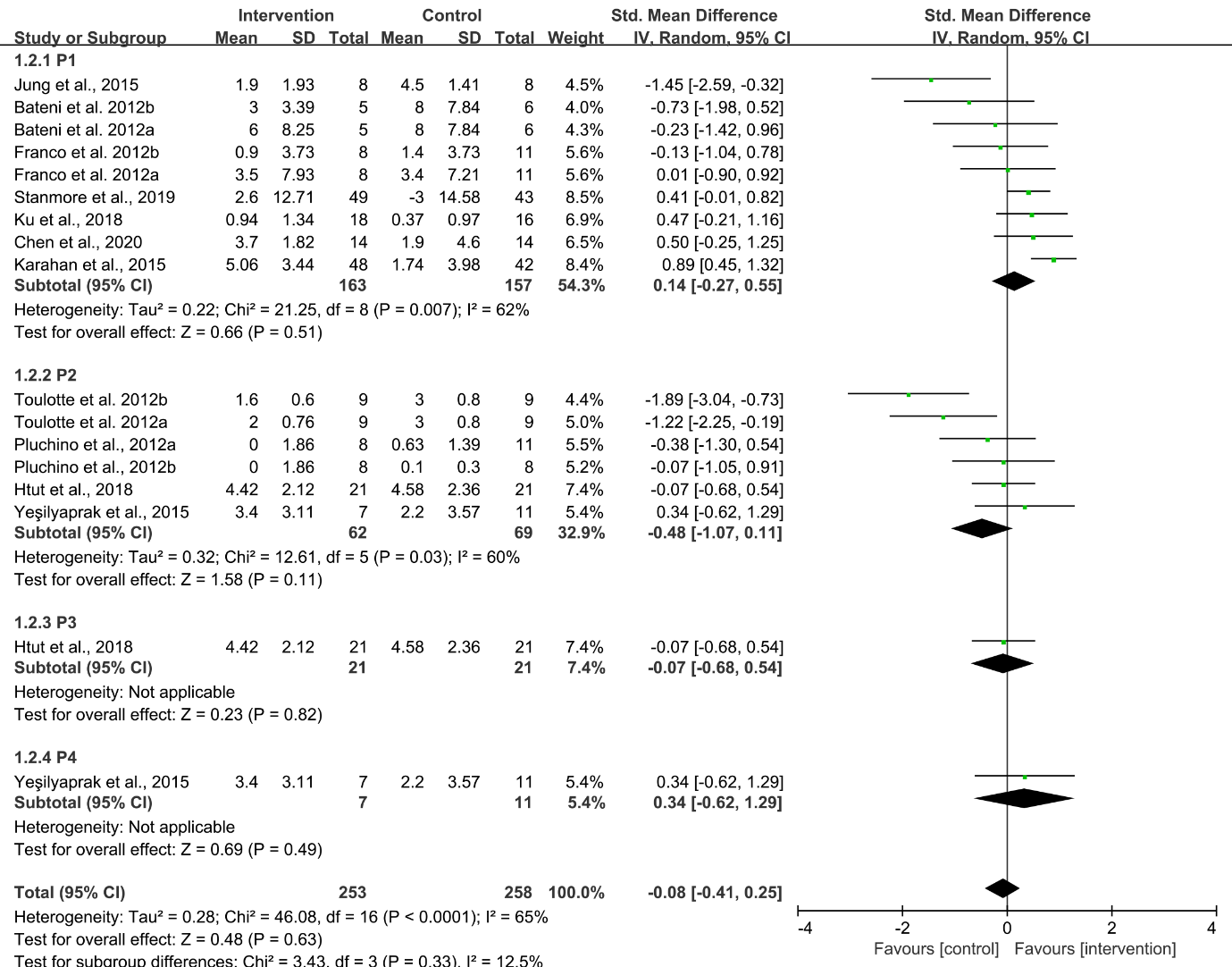


C


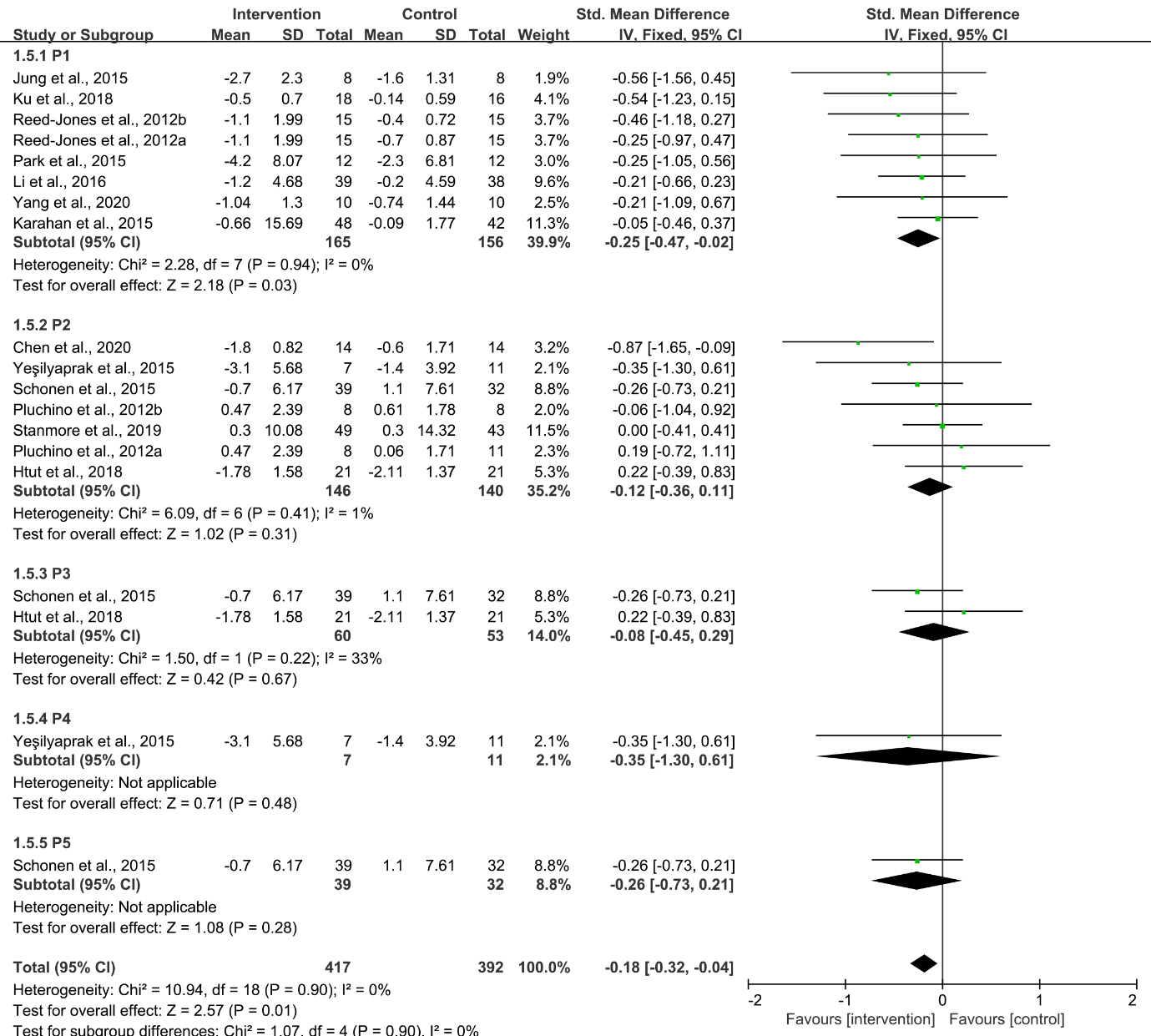


Figure S1. Result of subgroup meta-analysis by principles of motor learning. (A) Fall efficacy, (B) BBS, and (C) TUG. P1, learning occurs through repetitive, varied practice of meaningful tasks; P2, learning occurs when task difficulty is progressively increased according to the user's ability; P3, learning occurs when the individual is motivated to improve; P4, sensory feedback that is related to the task is necessary for learning; P5, learning occurs when an individual receives positive feedback about task performance and task accomplishment.
