# Supplementary material for: Comparison between the effects of exergame intervention and traditional physical training on improving balance and fall prevention in healthy older adults: a systematic review and meta-analysis
Source: J Neuroeng Rehabil. 2021 Nov 24;18:164. doi: 10.1186/s12984-021-00917-0 (PMC8611920; doi:10.1186/s12984-021-00917-0)
Supplement: Supplementary file 3 — Additional file 3: Figure S3. Result of subgroup meta-analysis by program period. (A) Fall efficacy, (B) BBS, (C) OLS, (D) FRT, (E) sway length, and (F) TUG. [file 12984_2021_917_MOESM3_ESM.docx]

A


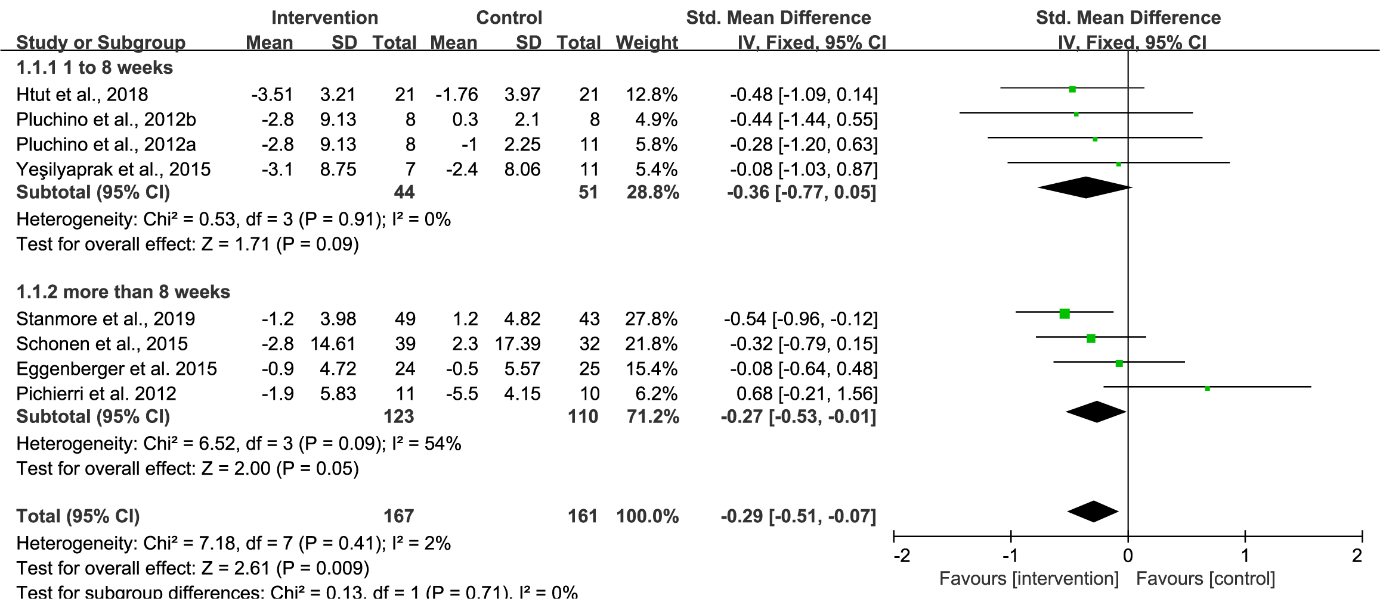


B


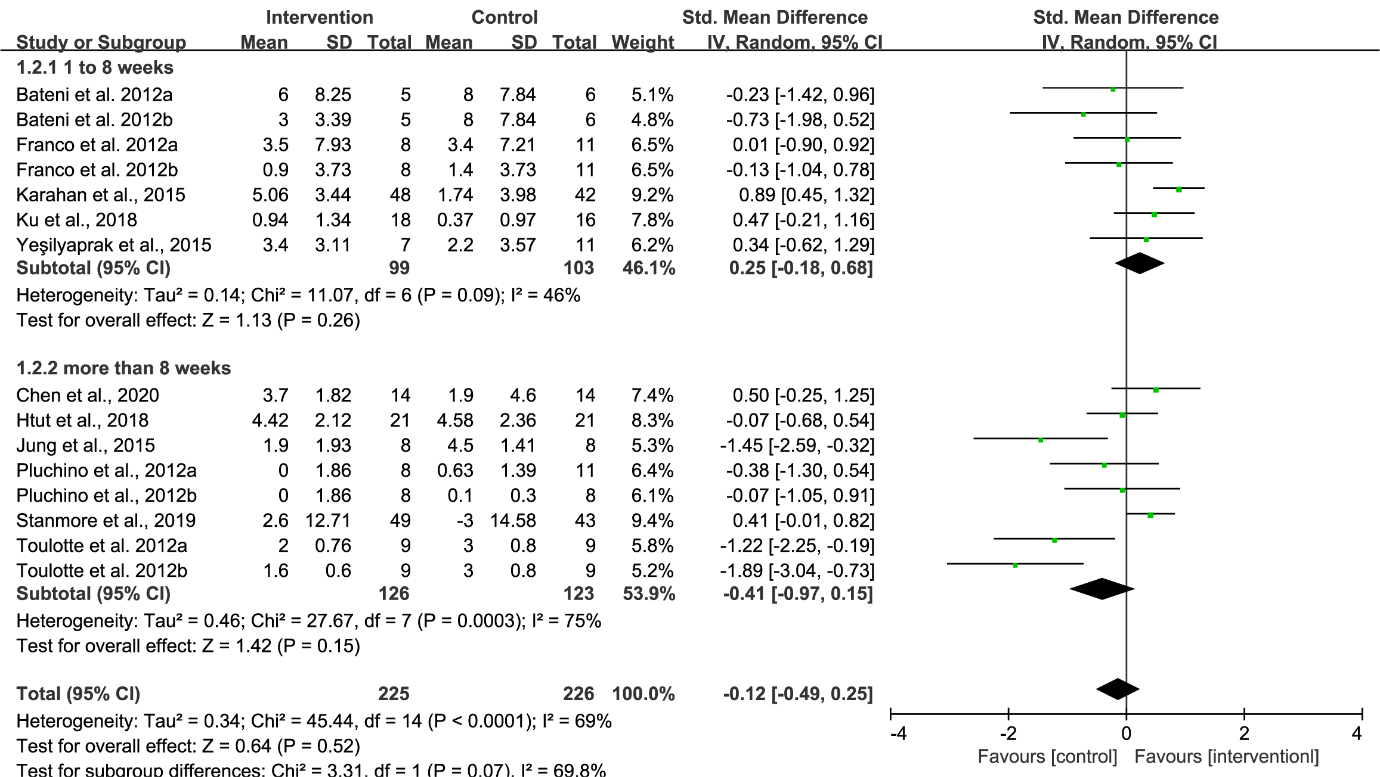


C


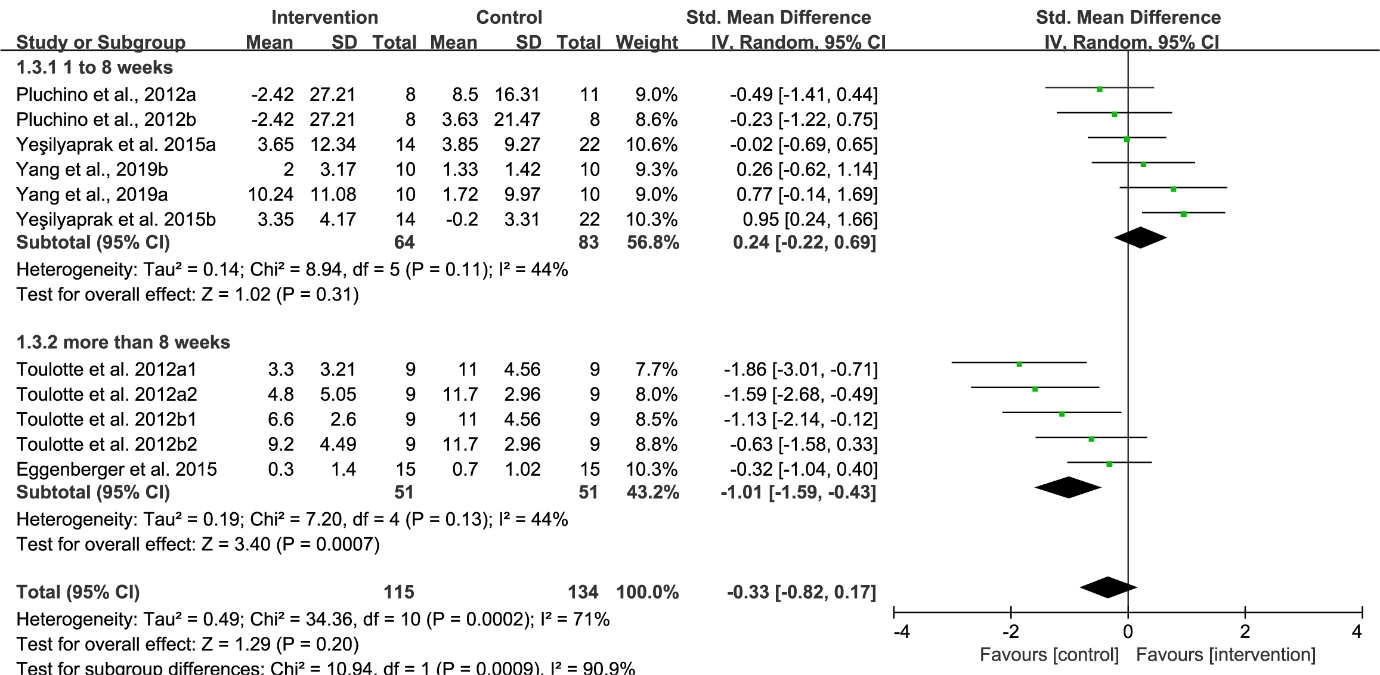


D


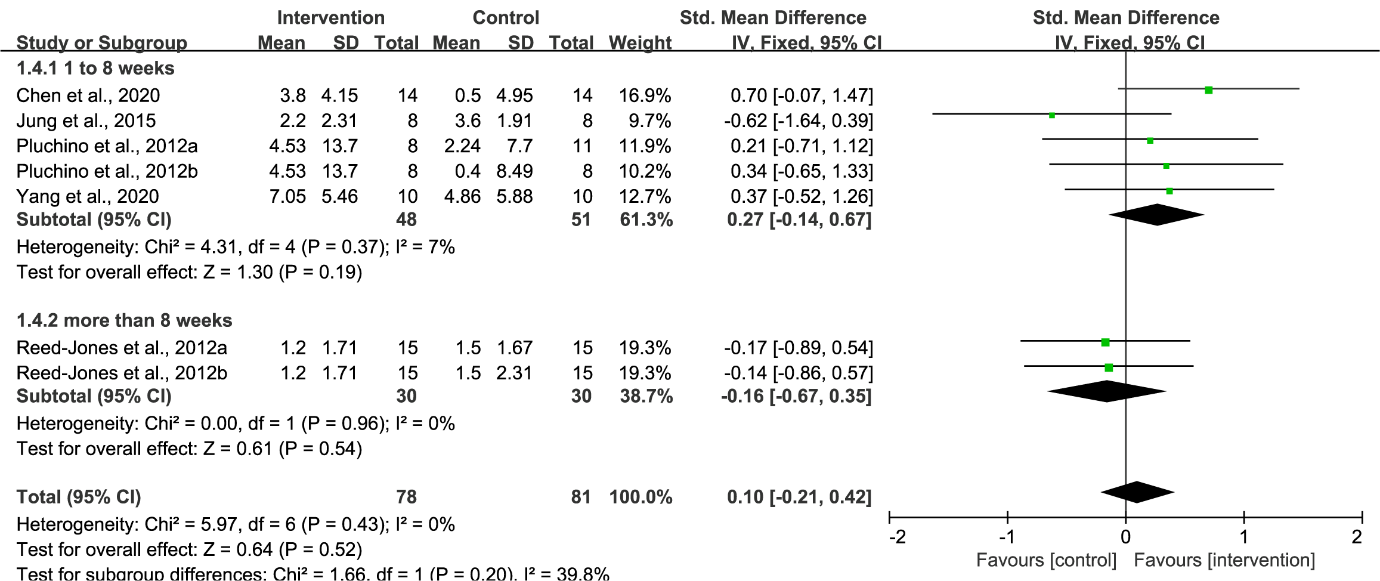


E


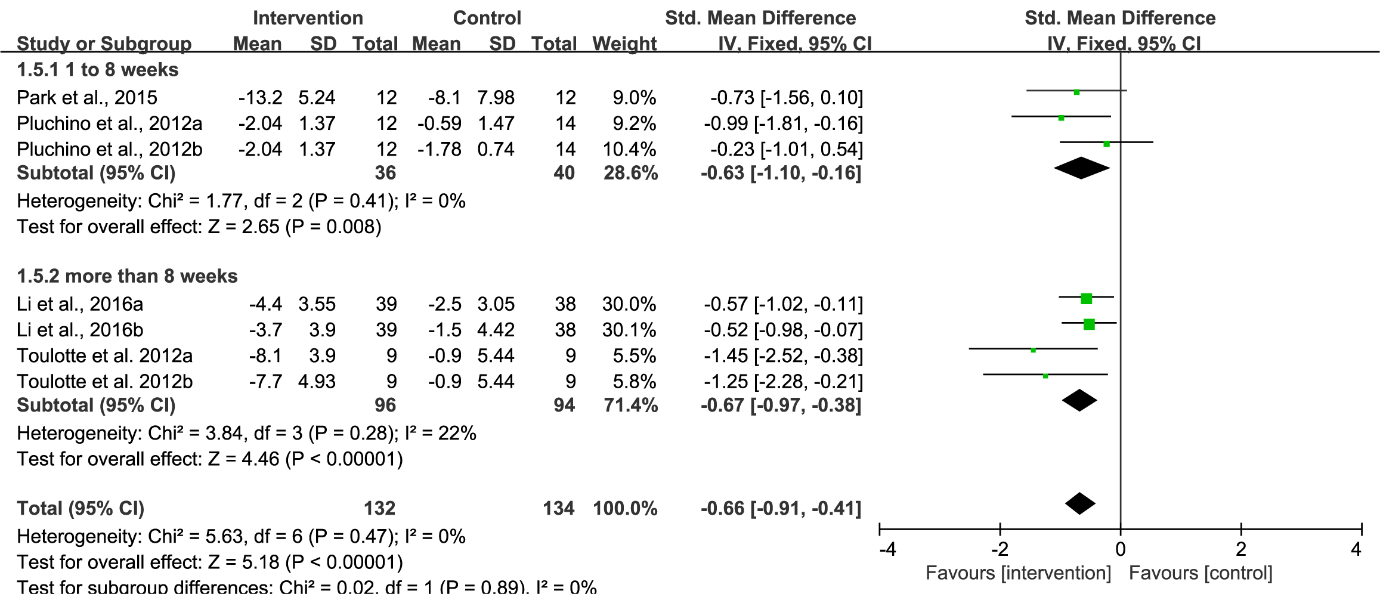


F


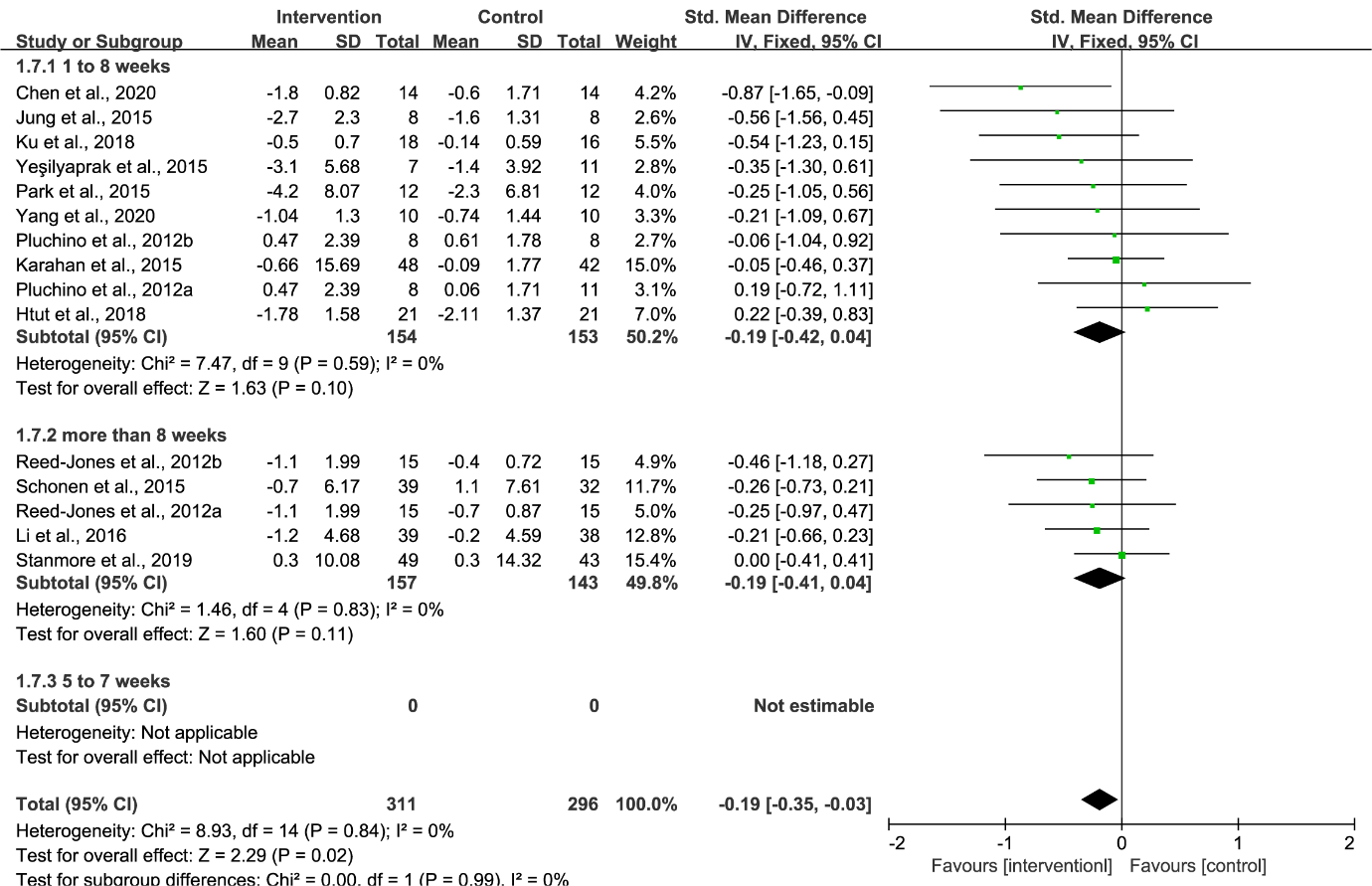


Figure S3. Result of subgroup meta-analysis by program period. (A) Fall efficacy, (B) BBS, (C) OLS, (D) FRT, (E) sway length, and (F) TUG.
